# Iatrogenesis as a possible cause of chronification of mental disorders

**DOI:** 10.1192/j.eurpsy.2021.500

**Published:** 2021-08-13

**Authors:** C. Martín Villarroel, L. Carpio Garcia, M. Sánchez Revuelta, J. Matsuura, G. Belmonte García, J. Dominguez Cutanda, M. Fernández-Torija Daza, E. García

**Affiliations:** Psiquiatría, Complejo Hospitalario Universitario de Toledo, Toledo, Spain

**Keywords:** iatrogenesis, chronification, Anxiety, therapeutic interventions

## Abstract

**Introduction:**

Most mental disorders tend to relapse (severe or mild pathologies such as anxiety or dystima disorders), which are potentially recoverable and yet, tend to evolve poorly, persisting residual symptoms without achieving a complete recovery.

**Objectives:**

The objective of this paper is to analyze the factors that influence process of recurrence and chronification, among which are our own therapeutic interventions.

**Methods:**

A bibliographic search was performed from different database (Pubmed, TripDatabase) about the iatrogenic potential of our intervention (psychopharmacological or psychotherapeutic), analyzing influence and mechanisms involved, and the way to prevent them.

**Results:**

Anxiety is a necessary element for the development of people, both from a biological perspective (natural and adaptive psychological response that allows us to respond adequately to possible threats); as an evolutionary psychological (element involved in conflict resolution, in turn necessary for personal development). It would be a mistake to consider it as pathological and try to eliminate it through medication or psychotherapy, since we could interfere with the natural recovery processes, contributing to its chronification and preventing possibility of change. At times, anxiety can be pathological when it occurs disproportionately and exceeds ability to adapt, but we must not eliminate it but to study origin and factors involved, to achieve complete resolution.

**Conclusions:**

In conclusion, we must consider possible iatrogenesis of our therapeutic interventions in process of chronification of mental disorders and try to avoid them by adequately studying individual factors and characteristics, before intervening.

